# Modelling strawberry quality in a longitudinal study under the marketing concept of branding

**DOI:** 10.1016/j.heliyon.2021.e06165

**Published:** 2021-02-15

**Authors:** Thais Mendes da Silva, Nicole Roberta Giuggioli, Cristiana Peano

**Affiliations:** Department of Agricultural, Forest and Food Sciences (DISAFA), University of Turin, Largo Paolo Braccini, 2, Grugliasco (TO), 10095, Piedmont, Italy

**Keywords:** Sensorial analysis, Texture analyser, Strawberry, Fruit quality, Brand, PLS model

## Abstract

**Background:**

Marketing strategies, such as branding, redefine how consumers perceive quality and create new requirements related to season length and quality homogeneity, among others. For short-day (SD) strawberry cultivar brands, the commercial season is short due to a dependency on temperature and photoperiod. A plausible strategy to extend the commercialization period is to use different varieties within a single brand; however, this has led to inconsistent quality in other fruit crops. A form of quality assessment to evaluate the impact of a multi-varietal brand on sensory quality is a critical longitudinal study with several sources of variability, such as the inherent variation among assessors and fruit replicates that can affect the reliability of the results. Therefore, this study aimed to develop a methodology to assess the sensorial and physicochemical quality of strawberry brands in two contexts: a short-term season composed of two SD cultivars and a long-term season with one SD and one day-neutral (DN) cultivar.

**Results:**

New statistical models are proposed in this study. An ANOVA mixed model with assessors and replicates as random terms and a multiple factor analysis highlighted a lack of homogeneity with regard to texture parameters and sourness, while partial least square models identified aroma and sweetness as the best quality indicators.

**Conclusions:**

This work has successfully illustrated a methodology that is capable of handling critical aspects of longitudinal studies by using univariate models that account for different sources of variability and constrained multivariate models to relate parameters with overall liking. A long-term brand is a viable solution to valorise strawberries, as parameter heterogeneity did not affect overall quality.

## 1 Introduction

The quality of horticultural products is often determined either by the intrinsic characteristics inherent to the nature of the product and by the extrinsic characteristics that are influenced by socioeconomic and marketing factors [1]. However, both intrinsic and extrinsic factors interact rather than complement each other given that new horticultural marketing strategies shape and redefine how the quality of a product is perceived by consumers, which then results in the formulation of new intrinsic quality requirements. Among marketing techniques, branding is a promising horticultural marketing strategy to boost the notoriety of a product and promote recurrent purchases throughout the season of that product. In this context, the length of the season and homogeneity with regard to perceived quality will unequivocally determine the success of a given product.

Among horticultural seasons, the strawberry commercial season is particularly short due to many factors. For example, the highly perishable nature of strawberries does not allow for storage over several months as may be done with apples or kiwifruits, while environmental conditions affect the fruit sets of some varieties, such as short-day (SD) cultivars in which the length of the fruiting season depends on the photoperiod and temperature [2]. One feasible strategy to extend the commercialization period of a fruit is to use different types of varieties within a brand; however, this has led to inconsistency with regard to the perceived quality of other fruit species [3]. Due to the dependency on photoperiod and temperature, many SD strawberry cultivars in Italy are cultivated in different territories, which can increase the heterogeneity within a given brand due to the different environmental conditions and agronomic practices employed during cultivation [4]. The use of day-neutral (DN) cultivars, which are insensitive to photoperiod, has increased in many production areas [5] and seems to be a promising strategy to extend the commercialization period of the product. However, in Italy, the availability of DN cultivars remains limited [2]. As a result, only single-cultivar brands are present in the marketplace (e.g. the Sabrosa cultivar of the Candonga® brand) with limited commercial seasons.

Consumers mainly purchase strawberries due to its high content on nutraceutical compounds [6], however, it is well known that a product recurrent purchase is also highly dependent upon an enjoyable eating experience [7]. In southern European countries, the sector has identified a reference criterion with regard to taste and aroma for SD cultivars, such as Sabrosa and Sabrina [2, 8, 9]. Many assessments have been carried out to evaluate and compare the quality of DN and SD cultivars; however, no study has investigated the perceived quality of both cultivars in a longitudinal study with a brand context. A quality assessment is often complex and involves the evaluation of several attributes, which are often highly collinear [10]. The investigation of the sensorial parameters between products that are harvested sequentially throughout the season is critical considering the uncertainty involved in human judgement [3], and other sources of variability such as the variability among replicates that is commonly present in horticultural products [11]. Therefore, the use of proper statistical techniques that are able to deal with multicollinearity and variability of the data is essential to obtain reliable results. Previous works [3] have shown that there are many multivariate techniques that are able to deal with multicollinearity, such as the multiple factor analysis (MFA) and more constrained techniques, such as the Partial least square (PLS) model, which is also able to put in relation a set of data with a different set of data, enabling the possibility of determine and quantify the relationship among parameters, just like the classic regression models.

Among the different statistical approaches, ANOVA (analysis of variance) mixed models have become widely used in the sensory field due to the possibility of introducing a within-group random factor that takes into account the variability between assessors and replicates [12]. This property is extremely important in sensory repeated measures designs where the inherent variation within assessors and replicates must be accounted to prevent their differences and/or errors from partially contributing to the error term of the model [13]. Finally, in this type of model, the compound symmetry structure of the model's covariance straucture may be changed to a more appropriate structure if required. This means that it is possible to set the correlation patterns of measurements taken over time. The assumption of compound symmetry implies that the correlation patterns are constant across the sensory sessions, even though this may be unrealistic since the judgments of multiple assessors might not change over time at the same rate for the same product. The measurements of a single assessor are presumed to be correlated, but the magnitude of those correlations is actually unknown [14].

Therefore, this study aimed to develop a novel methodology to assess the quality of horticultural brands. For strawberries in particular, the developed methodology is expected to provide information at three levels: 1) an evaluation of the impact of different strawberry cultivars on the sensorial quality of a given brand, 2) the identification of the parameters that could potentially affect the homogeneity of the brand, and 3) the identification of parameters that are superior quality indicators.

## 2 Materials and methods

### 2.1 Materials

Two quality assessments were carried out. The first assessment evaluated a short-term commercial brand (ST_brand) composed of two SD cultivars from southern Italy, while the second assessment evaluated a long-term brand (LT_brand) with an SD and DN cultivar from southern and northern Italy, respectively. The fruits were harvested when 75% of the skin surface was red. All brands were assessed with physicochemical and sensory analyses.

The SD_brand was composed of two cultivars (Sabrina and Melissa) from the same supplier in Campania (Latitude: 40° 36′ 31.578′′ N; Longitude: 14° 58′ 58.925′′). The brand was assessed weekly, and the varieties were sampled in random order due to the natural variation among fruit harvesting dates within the same period (March to May). Four samplings were carried out for each variety, which means four replicates were obtained for each variety. Each variety was assessed individually in 8 sensory sessions. The samples of the ST_brand were named Sabrina_Y1 and Melissa.

The LT_brand was composed of two samples: Sabrina (from the same southern Italian supplier from Campania as that of the SD brand), and Portola (a DN cultivar from a northern Italian supplier in Piedmont). The brand was assessed weekly, and the varieties were sampled in a set order due to the distinct harvesting periods of both varieties [i.e. Sabrina (March to May) and Portola (August to September)]. Five samplings were carried out for each variety, with 5 replicates for each variety). Each variety was assessed individually in 10 sensorial sessions. The samples of the LT_brand were named Sabrina_Y2 and Portola.

Both quality assessments consisted of longitudinal studies with replicates of each sample harvested and analysed over time. This was in line with the aim of this study, which was to evaluate the homogeneity between the two samples of the same brand by taking into account the variability within each sample over different sensorial sessions, represented by the replicates. Sabrina, which is one of the most commercialized varieties in Italy [9, 15], was chosen to be included in both brands as a reference for taste and overall quality.

### 2.2 Physicochemical analyses

Twenty fruits from each replicate were analysed for total soluble solids (TSS) with a PAL-1 digital refractometer (Atago, Tockio, Japan) according to Organization for Economic Cooperation and Development (OECD) guidelines [16]. The titratable acidity (TA) of the strawberry juice from 20 fruits was determined in triplicate for each replicate by titration with 0.1 N NaOH to pH 8.1 and expressed as g 100 g^−1^ of citric acid. The TSS: TA ratio was also calculated. Size was determined by measuring the maximum diameter of the equatorial section of the fruit.

#### 2.2.1 Texture analysis

Firmness was determined for the 20 fruits using a puncture test with an FTA 53220 fruit texture analyser (Turoni SRL, Forlì, Italya, 6-mm tip) according to OECD guidelines [16]. Considering that the assessment of strawberry texture is highly dependent on the type of method that is used, and variations on the results may occur whether it is applied a compression or a puncture test [17], the puncture test was complemented with a texture profile analysis (TPA) to evaluate the method and parameter best correlated with the sensorial parameter of hardness. The samples were compressed twice during the TPA with a TA.XT2+ texturometer (Stable Micro Systems, Surrey, U.K.) with a compression platen (diameter 75 mm). The analyses were conducted on cut fruits to limit the influence of size and shape. Twenty strawberries with the crowns removed were cut longitudinally in two halves, and each half was compressed at a pre-tested speed of 5 mm s^−1^, a test speed of 10 mm s^−1^, and post-test speed of 10 mm s^−1^. The distance travelled by the probe was 7.4 mm, and the trigger force was 5 g. The speed test was faster compared to those of previous studies [18, 19] to better simulate the mastication process. The distance chosen was determined based on trial and error and was an intermediate value to ensure that all samples that had different hardness perception scores would not show the same behaviour as that of a perfect elastic material due to the extremely low compression force. At the same time, the extremely high compression force that would lead to a complete breakdown of the samples with no possibility of measuring their cohesiveness was avoided. As in the study by Aday et al. [20], the parameters of the resulting force–time curve of hardness, springiness, cohesiveness, adhesiveness, gumminess, chewiness, and resilience were registered. Hardness can be defined as the force necessary to attain a given deformation; although, it is an absolute value that is extremely dependent on shape and specimen size and does not take into account how quickly the force peak is reached during the test. Fruits that are perceived to be harder break at a smaller deformation value and higher compression force compared to fruits that are perceived to be softer. Therefore, the Young module, which is measured as the slope of the first peak of the force over time, was also considered in the assessment. As suggested by a previous study [17], this parameter reflects sample stiffness and may provide a better indication of the perceived hardness.

#### 2.2.2 Colorimetric analysis

Considering the importance of colour in horticultural products, which usually supersedes the importance of either flavour or texture [21], colorimetric analyses were performed with a CR-400 colorimeter (Konica Minolta, Tokyo, Japan) in the equatorial zones of the most and least colourful sides of twenty fruits for each replicate. The L∗, a∗, and b∗ values were recorded with Konica Minolta software (Spectra Magic NX). From the L∗, a∗ and b∗ values, other colour indexes that have been previously evaluated were calculated using the following Eqs. (1), (2), (3), (4), (5), and (6) to enhance the sensitivity of the colour evaluation [22, 23, 24, 25]:

(1) $${\text{C}}^{*}\text{: }\sqrt{{\text{a}}^{2}\text{∗}+{\text{b}}^{2}\text{∗}}$$

(2) $${\text{h}}^{\text{∗}}\text{: }\tan^{-1}\frac{\text{b∗}}{\text{a∗}}$$

(3) CI: 1000×a∗/L∗×b∗

(4) MIC: L∗×a∗/b∗

(5) COL: 2000×a∗/(L∗×C∗)

(6) H_index: (180−h)/(L∗+C∗)

### 2.3 Sensory analysis

A sensory analysis was performed on all sample replicates. Fifteen panellists from Sata SRL (Alessandria, Italy) were selected and trained in strawberry sensory evaluation as recommended by International Standards Organization (ISO) 8586 [26]. All participants agreed to participate in the sensory tests and informed consent was acquired before performing the tests. For the overall liking assessment, the panellists were trained to use the Sabrosa variety as a reference standard, which is considered the reference variety for taste among modern Italian producers [2]. The analyses were done weekly between 3 and 5 PM. Two different continuous scales that were compliant with ISO 4121-2003 [27] were used. Firstly, a hedonic scale with “dislike extremely” at one end “like extremely” at the opposite end was used for the overall liking assessment. Secondly, a continuous intensity scale with “extremely low intensity” at one end and “extremely high intensity” at the other end was used to assess descriptive sensory attributes. The descriptive sensory analysis included hardness, sweetness, sourness, and aroma. Panellists were asked to not consider the aspect or the colour of the replicates. To ensure homogenization for each sample replicate, the assessors shared different parts of the same fruit, which were cut in halves, as suggested by Bavay et al. [11].

### 2.4 Statistical analysis

#### 2.4.1 ANOVA models

To fulfil the first goal of the assessment (i.e. to evaluate the homogeneity of quality within each brand), an ANOVA mixed model (model 1) was applied to the sensorial data using the 4 samples (Sabrina_Y1, Melissa, Sabrina_Y2, and Portola) and two brands (ST an LT) as fixed factors. The assessors and replicates for each sample were set as random factors to take into account the variation among scoring levels throughout the season [10, 28]. The five random terms derived from the interactions (sample: assessor; brand: assessor; sample: brand: assessor; brand: replicate; sample: replicate), which represent the assessor disagreements and replicate heterogeneity, were also introduced to the model and a stepwise deletion of model terms with high *p*-values was performed, as suggested by Kuznetsova et al. [28] to select only meaningful random effects and retain the most parsimonious model. A likelihood ratio test was used to assess random terms using an α level of 0.1, and the F-type hypothesis test was used for testing the fixed terms using an α level of 0.05, as suggested by Kuznetsova et al. [28]. Post-hoc analyses were conducted to evaluate the differences among samples, brands, and samples within each brand. The model was built with the R package “lme4” [29] and “SensMixed” [30]. Physicochemical data were analysed with a Student t-test was used to assess the differences among samples and brands. The results were compared to the sensorial data.

A second simplified ANOVA mixed model (model 2) was built using only the samples as a fixed effect and the assessors as a random effect to evaluate if the assessors gave different scoring rates throughout the commercial season for each sample. The model was built using the autoregressive covariance matrix AR (1) [AR (1) model] and was compared to the standard model using the compound symmetry covariance matrix (CS model). Three information criteria (AIC, Akaike's Information Criteria; BIC, Bayesian, and the *p*-value of the ratio of log likelihood values of both models) were used to determine which of the models best fit the data. These statistics are functions of the log likelihood and can be compared across models if the fixed effects of the model are constant. The likelihood-ratio test assesses the goodness of fit of two competing statistical models based on the ratio of their likelihoods. It tests whether this ratio is significantly different from one at an α level of 0.05 [31] and it was calculated as indicated in Pinheiro's work [32]. The models were built using the R package “nlme” [32].

#### 2.4.2 Multiple factor analysis (MFA) and partial least square (PLS)

A multiple factor analysis (MFA) and a partial least square (PLS) model were applied to fulfil the second and third goals of the assessment, i.e. to identify the parameters that may potentially affect quality homogeneity of the brands and identify the best quality indicators, respectively. The MFA analysis was used to identify which parameters contributed most to differences among samples and their replicates. The MFA builds a common space based on the dimensions that explain the maximum common variability of a set of parameters [33] measured from the same replicates. The twenty-five parameters assessed were scaled and grouped into new active continuous sets of variables named “instrumental taste” (TSS, TA, and the TSS: TA ratio), “colour” (all colorimetric parameters), “instrumental texture” (firmness and all TPA parameters), and “sensorial” (all descriptive sensorial parameters with the exception of overall liking). Two supplementary categorical groups (i.e. “sample” and “brand”) were added to respectively group replicates by the Sabrina_Y1, Melissa, Sabrina_Y2, and Portola samples, the Sabrina, Melissa, and Portola varieties, and the two brands (ST and LT) to obtain the replicate centroids. Considering that the Overall liking parameter was the result of the interaction of the quality parameters determined by the panellists, it was also added as a supplementary continuous variable. The supplementary groups were only introduced to facilitate the interpretation of the analysis [34] and had no influence on the common space created. Significant differences that were detected among samples and brands were assessed using ellipse confidence intervals at an α level of 0.05. A scree plot was produced to determine the number of dimensions to retain in the model.

Finally, to fulfil the third goal of this assessment, a PLS regression model was built to highlight potential quality indicators, as described by Mendes da Silva et al. [33]. In particular, in this study, the PLS model, which was built with the package “plsdepot” [35], was used to provide a quantitative estimation of the relationship between the single dependent variable Overall quality and the independent variables, represented by all parameters assessed in the MFA analysis. All parameters were introduced as continuous data and scaled. The explained variance (R^2^) of the model was assessed, and a cross-validation process was used to determine how many dimensions to retain in the model. It is well known that the predictive capacity of the model increases when more components are included; however, this approach may introduce variation to the data that is not explained by the model and that is attributed to noise, and the model can suffer from over-fitting [36]. Therefore, the prediction residual sum of squares (PRESS) was assessed to choose the proper number of components [37], along with the cumulative function of the Q^2^ index, which indicates the explained variance of the testing data derived from the cross-validation step. This index is expected to decrease at some point due to the introduction of non-systematic variance by the addition of a new component [36]. The loading weights, which represent the effective loadings directly connected to the construction of the regression relationship between predictors and the dependent variables instead of the p-loadings [38], were assessed in order to evaluate which parameter contributed most to overall liking. While the p-loadings indicate the correlation values among parameters and dimensions without taking into account the relationship between the dependent variables and the dimensions, the loading weights indicate the correlation values of a model that take into account the constrain of building dimensions maximizing either the differences among samples as well as the correlation between the dependent variables of the model and its dimensions. This is the reason why it is possible to build a space where the overall liking is better explained, being the latter the dependent variable of the model.

## 3 Results and discussion

### 3.1 Sensorial evaluation of samples and brands

#### 3.1.1 Sensorial evaluation (model 1)

To fulfil the first goal of this assessment, which was to identify which sensorial parameters were sources of heterogeneity throughout the commercial season of both brands, a simplified ANOVA model for each sensorial parameter was built using only significant random terms to evaluate differences among the fixed effects (samples and brands). The selection of significant random terms is needed to build models that are less specific and more generalizable, as suggested by Kincaid [31]. The results of the stepwise selection procedure are shown in Table 1. The random terms Assessor and Samples: Brands: Replicate were significant (p-value < 0.1) for most of the sensorial parameters, which indicated they should be retained in the parameter models [28]. Overall liking was the parameter in which the analysis detected the highest number of significant random terms, which included the term, Samples: Brands: Assessors, while sweetness and aroma only presented the term Assessors as significant.

**Table 1:** *p*-values of the likelihood ratio test results of all sensorial parameters for the random-effects.

Random terms	*p*-values of likelihood ratio test				
	Overall liking	Hardness	Sweetness	Sourness	Aroma
Samples:Assessors	1.000	0.996	1.000	1.000	1.000
Samples:Replicate	0.997	1.000	1.000	0.999	0.999
Brands:Assessors	0.603	1.000	0.523	0.903	0.714
Brands:Replicate	0.446	1.000	0.469	1.000	1.000
Brands:Assessors:Replicates	0.331	0.667	0.997	0.997	1.000
Samples:Brands:Assessors	0.019	1.000	0.544	0.321	0.419
Samples:Brands:Replicates	0.013	0.000	1.000	0.001	0.175
Assessors	0.057	0.002	0.055	0.232	0.070
Replicates	1.000	1.000	1.000	1.000	1.000
Assessors:Replicates	1.000	1.000	0.995	1.000	0.864

As expected, results from Table 1 suggest that there were variations among assessor scores for almost all parameters, while a disagreement between assessors was only found for the overall liking scores given for samples within the same brand. Replicates of samples within the same brand also presented significant variation with regard to the overall liking, hardness, and sourness scores throughout the season. In addition, for most of the parameters, the p-value was lower than the assessor disagreement term. This suggests that the replicate term in the variance component was larger than the disagreement among the Assessor terms, meaning that the contribution of the replicate to the variance was larger than that of any other contribution. Therefore, taking into account the replicate variance over time was important to the assessment.

**Figure 1:**
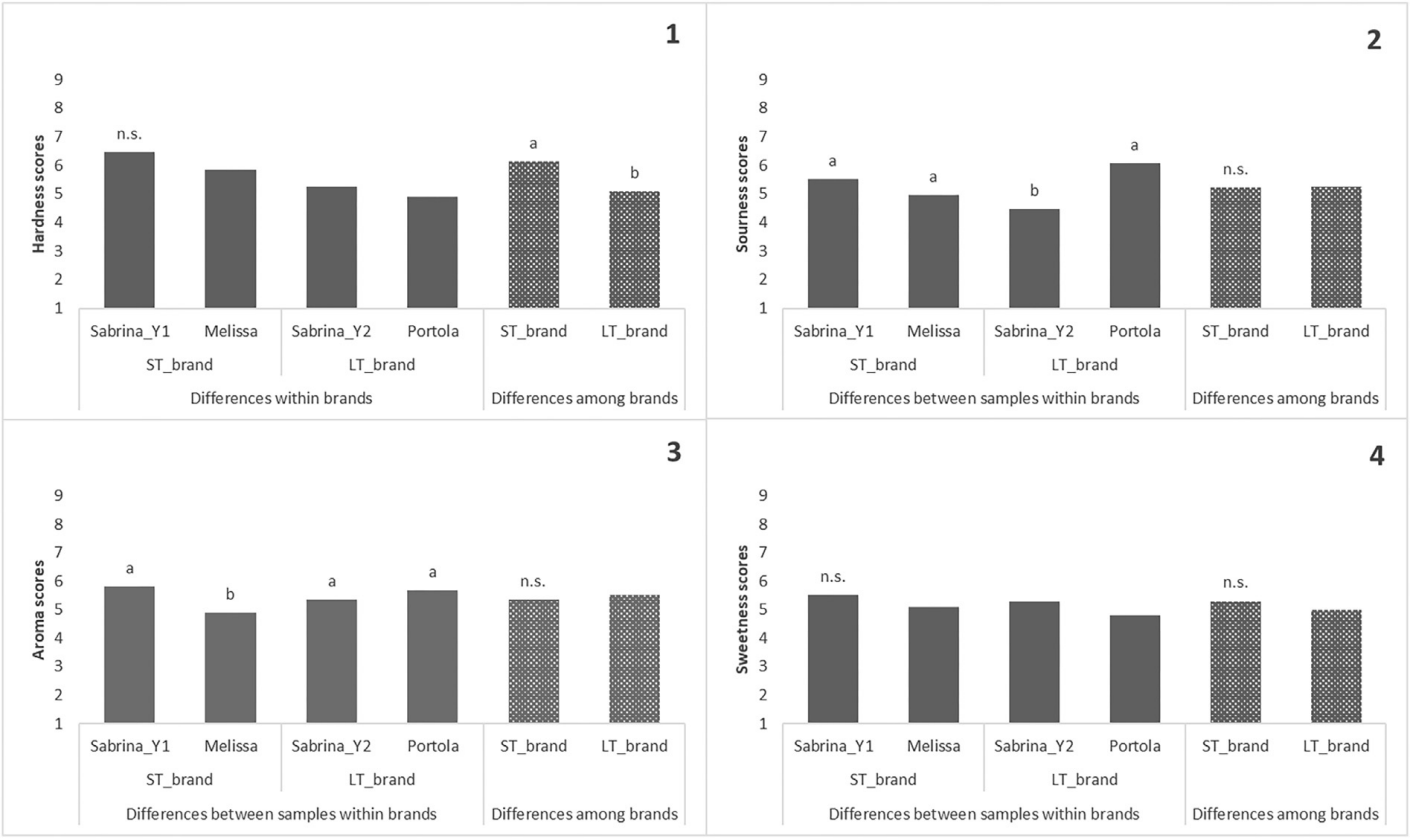
Post-hoc ANOVA results of model 1 for hardness (1), sourness (2), aroma (3) and sweetness (4) scores obtained from the sensory analysis of the Sabrina (Sabrina_Y1) and Melissa strawberry samples within the ST_brand, the Sabrina (Sabrina_Y2) and Portola samples within the LT_brand, and the overall samples among ST and LT brands. Different lower-case letters (a–b) show significant differences among samples and brands (p-value ≤ 0.05), and n.s. indicates non-significant differences among samples and brands (p-value ≤ 0.05).

In Figure 1, it can be seen that there were overall significant differences between brands concerning Hardness, with the LT brand presenting lower values than those of the ST brand. In addition, there were significant differences among samples within the LT_brand with regard to Sourness. Finally, significant differences were present among samples within the ST brand with regard to Aroma. However, globally and within the brand, there were no significant differences with regard to Sweetness or Overall liking (data not shown).

Within the LT_brand, assessors perceived Portola to be sourer than Sabrina, while within the ST_brand, Melissa was considered less aromatic than Sabrina. In contrast to previous studies [39], most of the sensorial results agreed with physicochemical data in this study (Table 2), with the exception of firmness, which did not show any significant differences among samples or brands. The higher values of perceived Hardness registered for the ST brand were not expected since it has been found that ST cultivars are usually softer than DN cultivars [40].

**Table 2:** Student t test results of size, Total soluble solids (TSS), titratable acidity (TA) and firmness obtained from Sabrina (Sabrina_Y1) and Melissa strawberry samples within the ST_brand, the Sabrina (Sabrina_Y2) and Portola samples within the LT_brand, and the overall samples among ST and LT brands. Different lower-case letters (a–b) show significant differences among samples and brands (p-value ≤ 0.05), and n.s. indicates non-significant differences among samples and brands (p-value ≤ 0.05).

	size (mm)		TSS (°Brix)		TA (as g 100 g^−1^ of citric acid)		firmness (kg/cm^2^)	
Melissa	37.20	n.s.	6.78	n.s.	0.68	n.s.	0.63	n.s.
Sabrina_ST	35.80		7.60		0.71		1.11	
Sabrina_LT	40.00	a	7.52	n.s.	0.64	b	0.92	n.s.
Portola	22.80	b	6.70		0.84	a	1.35	
ST_brand	36.46	n.s.	7.19	n.s.	0.70	n.s.	0.87	n.s.
LT_brand	31.40		7.11		0.74		1.14	

There are many genotypic traits that might affect texture perception such as the arrangement and packing of parenchyma cells, morphology of cell walls with regard to pectin and fibrous polysaccharides, cell turgor, and cell wall adhesion. Moreover, texture properties are also affected by size and specimen [17], which are strongly related to genotype in strawberries. As it was described in Materials and Methods, in this work, each sample's replicate was only cut in halves, replicates were not cut in small pieces with the same length, width and specimen. Therefore, the preparation was probably only able to limit the replicate's variation but not to break down the variability completely among different variety's replicates. Varieties with different shapes could still have influenced the sensorial perception depending on their specimen and size. In fact, all Portola replicates presented a smaller average size (data not shown), which could have resulted in different hardness perception in this study. It is also possible to note that between Portola and Sabrina_LT samples there was probably a high variability among replicates as the statistical test did not point out any significant differences even though the firmness mean values among both samples are very different.

These results confirm that Sabrina presented better quality attributes than those of the other cultivars considering different sensory parameters, although these differences did not lead to a significant heterogeneity among Overall liking scores, even within the LT_brand, which was more susceptible to variations in quality considering the different origins of the product and physiology of the cultivars. These results are different from those of Mendes da Silva et al. [3] for multi-varietal apricot brands, in which different cultivars were also found to lead to different liking scores, and show how important it is to consider the specific quality perceptions of each fruit species to formulate a proper marketing plan for a given brand.

#### 3.1.2 Sensorial evaluation (model 2)

ANOVA model 2 was built using a compound symmetry covariance structure and autoregressive structure to evaluate if variations in the assessor scores displayed different patterns over time. This assessment is important to evaluate if the first goal of the brand quality assessment can be further improved by taking into account how variation within an assessor changes over time. It has been suggested that neglecting the complexity of the covariance structure by selecting the simplest one may increase the type error 1 rate of fixed effects [31], meaning that the null hypothesis (i.e. no significant differences among samples) might be rejected when it should be accepted. As demonstrated in Table 3, the ratio of the log likelihood values of the Overall liking scores was significant and indicates that model 2 improved when the AR (1) covariance matrix was used. The same was found for the Hardness and Sourness scores. A model with the AR (1) covariance matrix presents random terms (assessors) with correlations that decline over space or time [31]. This means that scores that are given over time by each assessor are correlated to each other (since they come from the same panellist over time), however, scores from samples that were assessed in closed sessions were more correlated than scores from samples that were far apart from each other in time. This is due to the fact that in the AR (1) covariance matrix, the degree of correlation between two observations (or residuals) is proportional to the relative amount of elapsed time and reduces exponentially. Despite the improvement obtained, both the CS and AR (1) models in this study led to the same results for model 1 for all attributes when post-hoc analysis was performed.

**Table 3:** ANOVA table of the model of the overall liking scores and the information criteria parameters of the Akaike's Information Criteria (AIC), Bayesian (BIC), Log likelihood, ratio of the CS model and AR (1) model log likelihood, and the *p*-value of the ratio of log likelihood values.

Overall liking scores ANOVA Model 2	AIC	BIC	Log likelihood values	Ratio of log likelihood values	*p*.value
CS model	797.915	817.94	785.9152	7.524927	0.0061
AR (1) model	792.390	815.75	778.3902		

### 3.2 Identification of parameters that affect brand homogeneity

**Figure 2:**
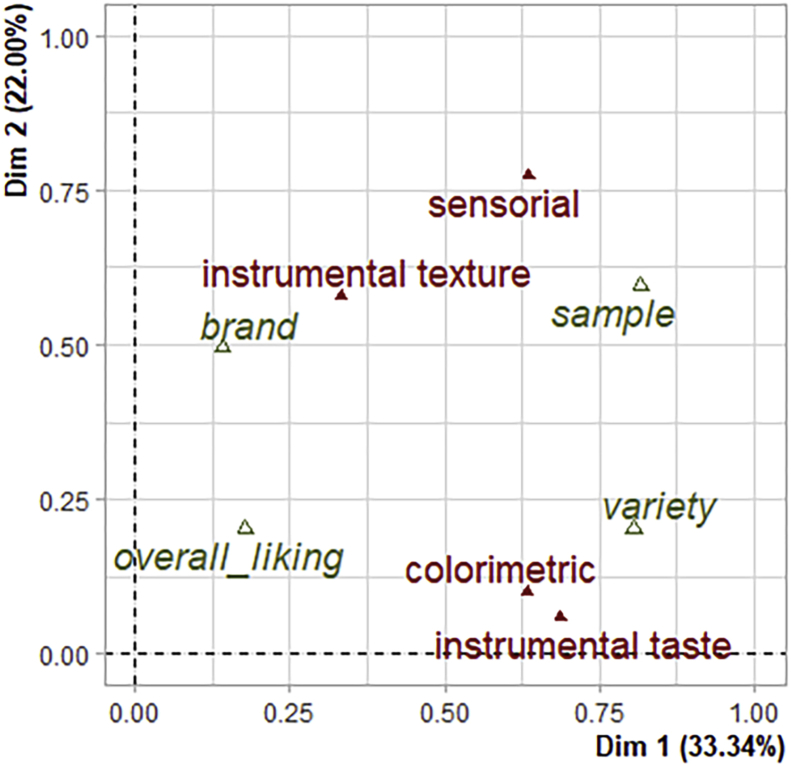
Biplot of the sets of variables on dimensions 1 (Dim 1) and 2 (Dim 2) after analysis of the physicochemical parameters (represented by “instrumental taste”), colorimetric parameters, texture parameters (represented by “instrumental texture”), and sensorial attributes of all strawberry samples. Active groups (“instrumental taste”, “colorimetric”, “instrumental texture”, and “sensorial variables”) are represented by red colour, while supplementary variables (overall liking, brand, variety, and sample) are represented by green colour.

An MFA was carried out to fulfil the second goal of the assessment, which was to identify the parameters that contributed most to the variability of a brand and that were potential sources of heterogeneity for the quality of the brand. A scree plot indicated that the first two dimensions accounted for most of all meaningful variance among the sample's replicates, with both presenting almost 60% of total variance explained (55,34%). This means the first 2 dimensions are summarizing more than half of the variability among the samples. The MFA contribution plot (Figure 2) shows that the instrumental taste, colorimetric, and variety groups were well correlated with the first dimension, while the instrumental texture and brand groups were more correlated with dimension 2. Sensorial parameters and sample groups were well correlated with both dimensions. Therefore, sensorial parameters seem to be important for discriminating among samples, brands, and varieties, while the instrumental texture group was able to particularly discriminate the two brands, and the colorimetric and instrumental taste groups were better able to discriminate among varieties. However, the supplementary parameter of overall liking was not well explained in either of the first two dimensions. Therefore, in this study, many of the parameters that impact homogeneity of quality are actually poor indicators of overall liking.

**Figure 3:**
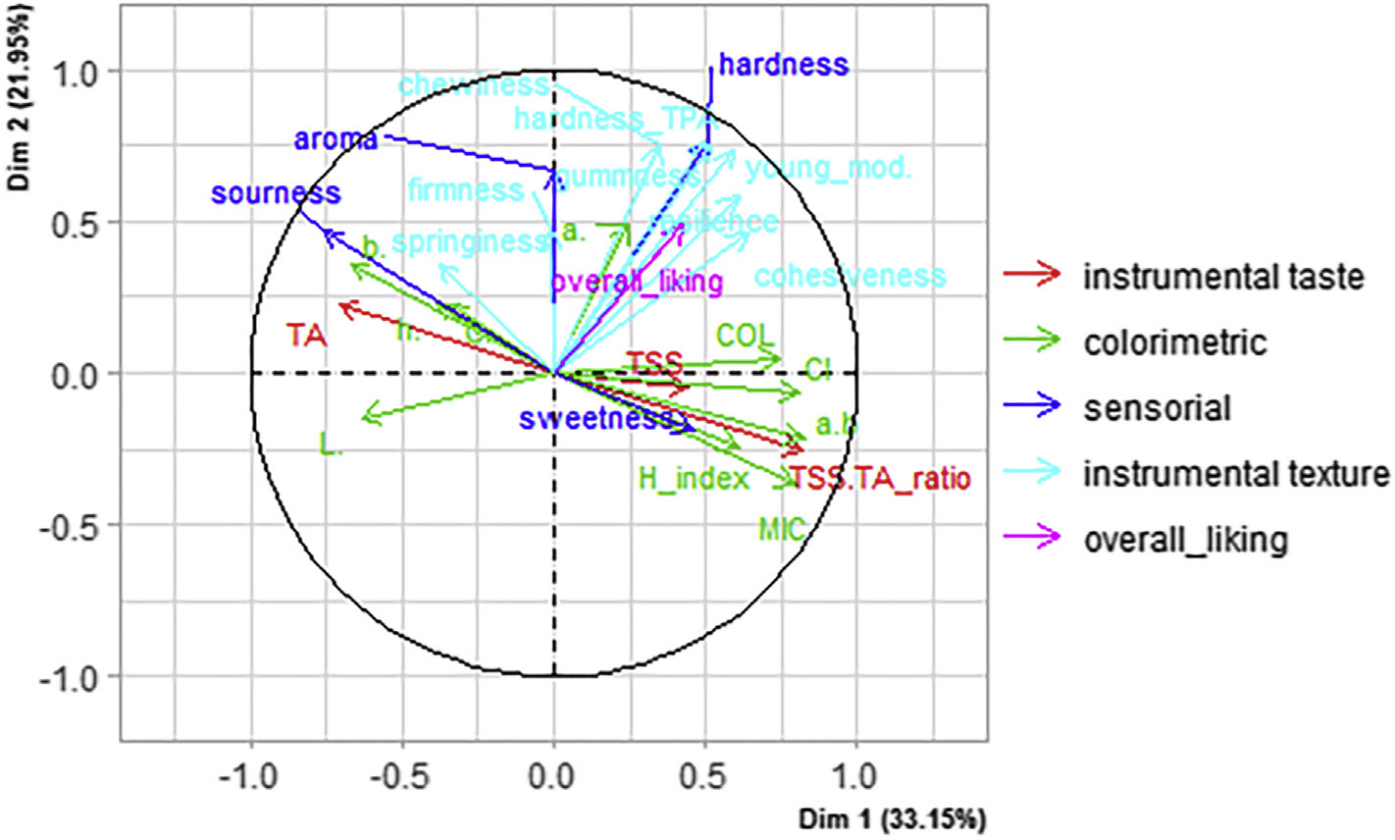
Biplot of physicochemical, colour, and sensory original variables on dimensions 1 and 2 after the analysis on total soluble solids (TSS), titratable acidity (TA), the TSS: TA ratio, the colorimetric parameters L∗, a∗, b∗, C (Chroma Eq. (1)), h∗ (hue angle, Eq. (2)), the composite colorimetric indices CI (Eq. (3)), MIC (Eq. (4)), COL (Eq. (5)), and H_index (Eq. (6)), the texture parameters: firmness, hardness_TPA (hardness measured with the TPA test), young mod (young module), cohesiveness, springiness, gummness and chewiness parameters, and the sensorial attributes of the strawberry samples.

By looking at the biplot of the parameters (Figure 3), with the exception of overall liking, sweetness, TSS, firmness, the colorimetric parameters a∗ and C∗ (Chroma, Eq. (1)), and springiness were poorly represented in the map. Therefore, those parameters should not be taken into account in the MFA interpretation.

By looking at Figure 3, it is still possible to evaluate the correlations among physicochemical, colorimetric (using Eqs. (1), (2), (3), (4), (5), and (6)) and TPA parameters with the descriptive sensorial parameters. Many TPA attributes were well correlated with the perceived hardness of the products at very similar rates, especially the parameters hardness_TPA (first peak in force from the force vs time curve) and gumminess. The fact that gumminess displayed a higher correlation with perceived hardness than chewiness agrees with what has been reported in the literature. It has been suggested that this parameter is more appropriate for measuring the hardness of soft food [41], such is the case with strawberries. This suggests that the current trend of describing new strawberry varieties as crunchy by breeders [42], which is an attribute that is generally associated with hard foods that tend to undergo fracture during mastication [43], might be misleading and is not actually perceived by consumers. The Young module seemed to display very similar behaviour to that of the other TPA parameters and was less informative than the hardness_TPA parameter regarding the relationship with perceived hardness. This agrees with the results of Gunness et al. [19], who found that the correlation between stress and the Young module was positive and high, even though the stress values were more discriminative than those of the Young module. It is likely that the use of the Young module to evaluate soft food is less important than its use when evaluating hard food, as has been suggested in a previous study [44].

The colorimetric parameters that indicate a tendency for red colour were all highly correlated among themselves and negatively correlated to the L∗ parameter. The use of the L∗ parameter to indicate the whiteness or brightness of horticultural products is widespread [23], even though both parameters can represent different quality attributes, with the former being associated with skin hue and the latter associated with an indication of freshness. Therefore, it is important to identify the real causes of the variations in L∗ values to avoid misleading interpretations. One of these causes may be attributed to the shape of the colour space. Since the eye does not equally detect differences in hue, chroma, or lightness [45], visual perception is actually represented by an ellipsoid. Therefore, each colour will present a viable range of L∗ values, meaning that red products will usually present lower L∗ values than pink products. Therefore, we suggest that only chroma values should be used to describe the brightness of a colour when evaluating strawberries. In this work, L∗ mainly reflects the lightness of the products. Both the TA and sourness parameters are negatively correlated with the tendency for red colour; however, considering that strawberries are a non-climacteric fruit and that replicates were analysed at a similar ripening stage, the tendency to red colour in this study was probably mainly linked to varietal characteristics [21].

**Figure 4:**
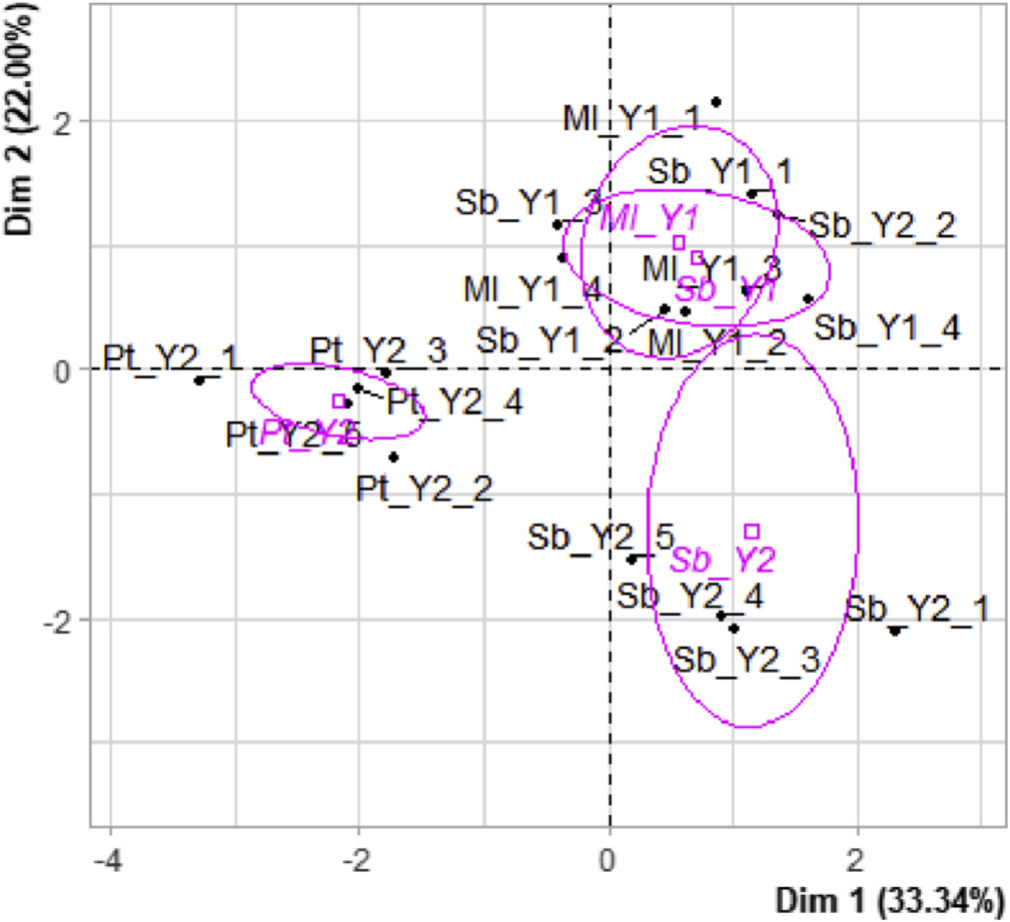
Biplot of the scores and confidence ellipses plotted on the first 2 dimensions (dim 1 and dim 2) after the analysis of total soluble solids (TSS), titratable acidity (TA), the TSS: TA ratio, all colorimetric parameters, firmness, TPA parameters, and the sensorial attributes of the strawberry samples. The 95% confidence intervals were calculated around the centroids of the factor scores for the Sabrina and Melissa samples from ST_brand (Sabrina_Y1, Melissa_Y1), and Sabrina and Portola samples from LT_brand (Sabrina_Y2 and Portola_Y2).

**Figure 5:**
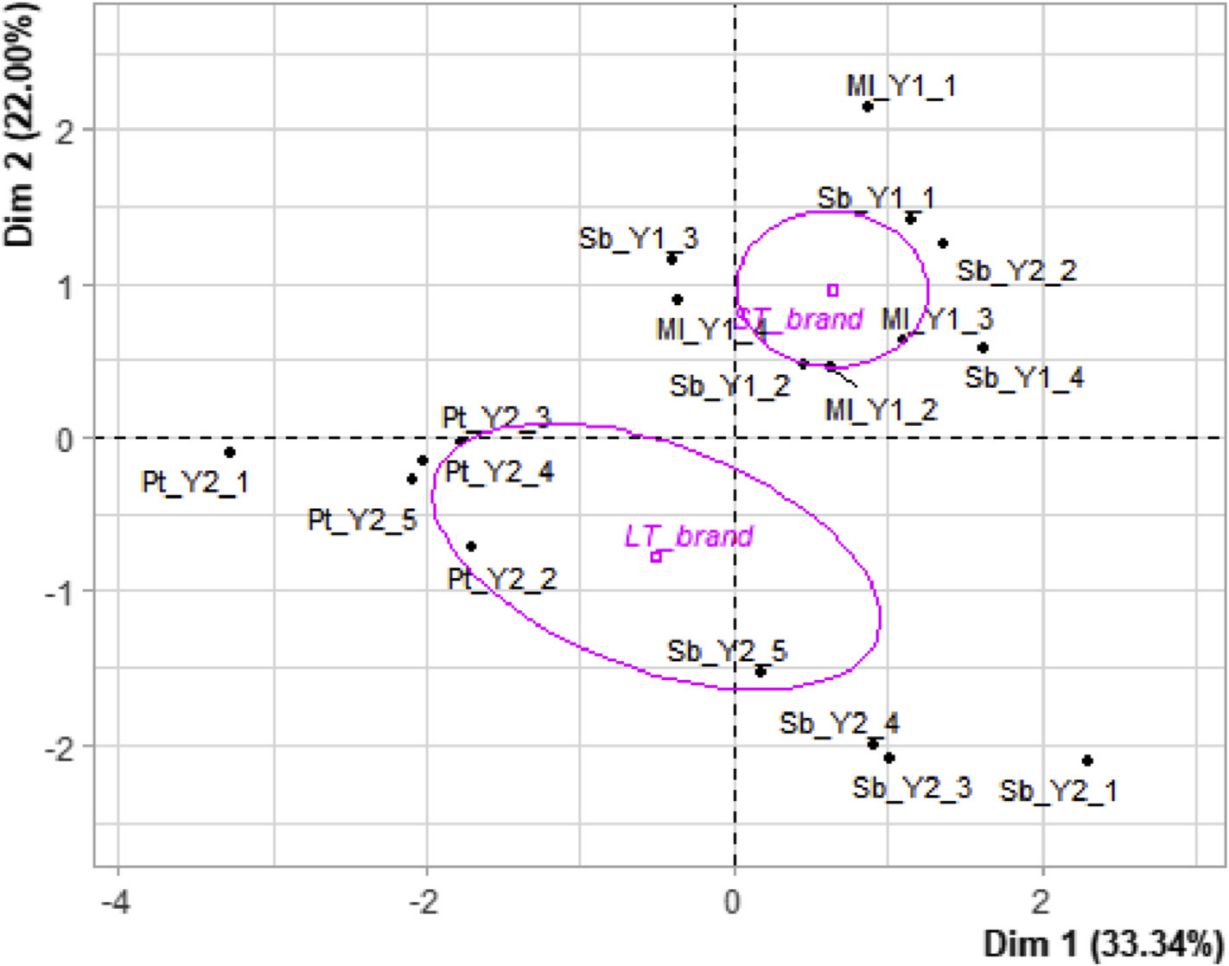
Biplot of the scores and confidence ellipses plotted on the first 2 dimensions (dim 1 and dim 2) after the analysis of total soluble solids (TSS), titratable acidity (TA), the TSS; TA ratio, all colorimetric parameters, firmness, TPA parameters, and the sensorial attributes of the strawberry samples. The 95% confidence intervals were calculated around centroids of the factor scores for the short-term (ST_brand) and long-term (LT_brand) cultivar brands.

As expected, in Figure 4 and Figure 5, it was possible to observe that the Melissa replicates were much more similar to the Sabrina replicates than to the Portola replicates (due to the centroid proximity) despite the brand context. The ST_brand, represented by the Melissa and Sabrina_Y1 samples, can in fact be considered very homogeneous under all parameters assessed, as was highlighted by the overlapping regions displayed by the Melissa and Sabrina_ST ellipses.

### 3.3 Identification of parameters that affect overall liking

**Figure 6:**
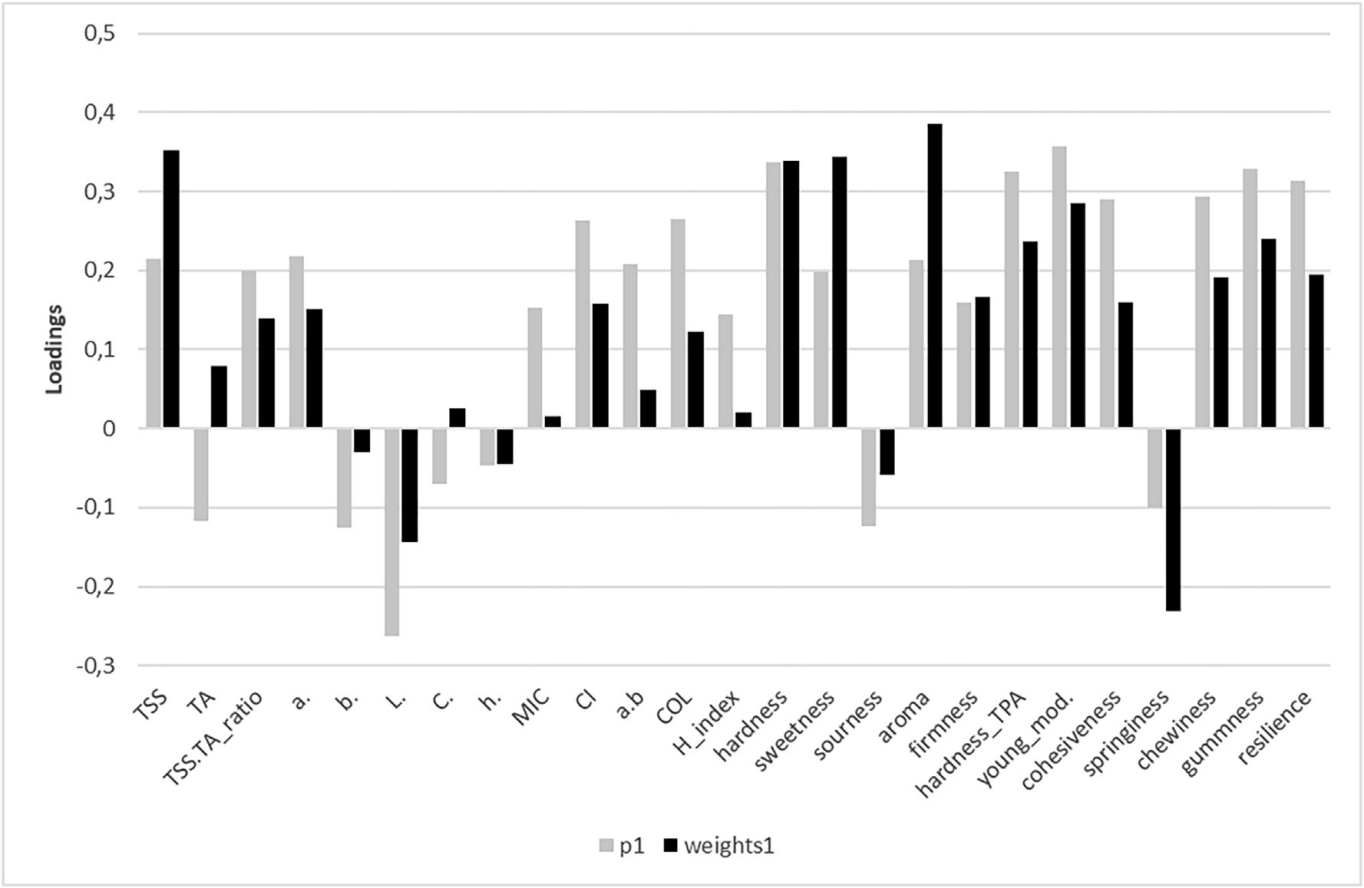
Plot of the parameter p-loadings (p1) and loading weights (weights1) of the PLS model.

Due to an inability of quantifying the relationships among parameters and the overall liking descriptor in the MFA analysis, a PLS model was created to constrain the correlations among the early dimensions of the model and the dependent variable (overall liking) as much as possible while also being explanatory of the predictor block. The PLS model is a compromise between a multiple regression of the dependent variable on the independent variables and a principal component analysis of the predictors [46], where the dependent variable structure guides the decomposition of the predictor matrix [38]. Therefore, by balancing both the dependent variable and the predictor information in the dimension construction, the PLS reduces the influence of large variations in the predictor block that do not correlate with the dependent variable structure. In this assessment, the model retained only the first two dimensions, with the first dimension explaining most of the variance of the predictors (R^2^ of 0.59) and the second dimension (R^2^ of 0.25). The Q^2^ index decreased from 0.55 to 0.48 after the introduction of a third dimension, suggesting that only the first two dimensions should be retained in the model. By comparing the loading weights and the p-loadings of the first dimension in Figure 6, it is possible to observe that for some variables, such as TSS, aroma, and springiness, the values varied depending on whether the first dimension was constructed under the constraining influence of the dependent variable (weights1) or not (p-loadings p1).

**Figure 7:**
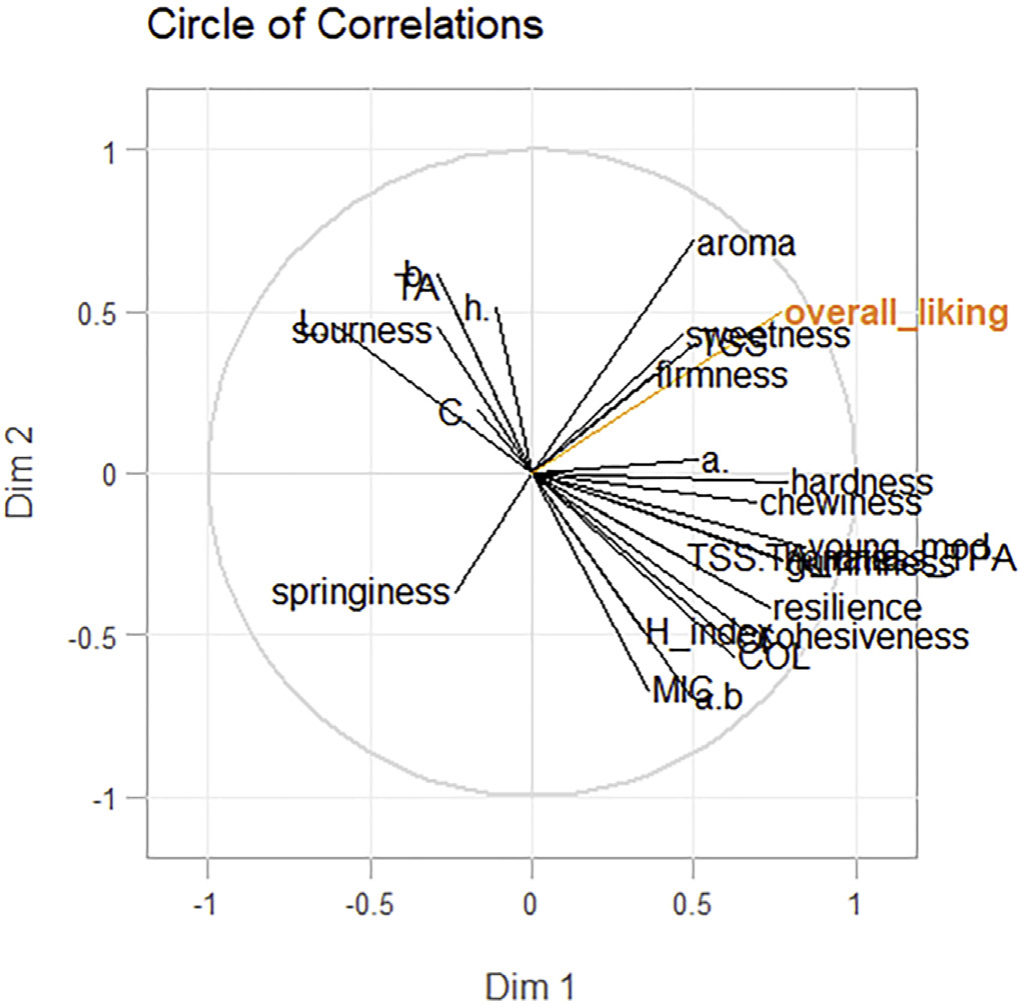
Plot of the PLS variables on the first two predictor dimensions (Dim 1 and Dim 2). The orange colour indicates the dependent variable, while the independent variables are indicated with black colour.

In Figure 7, the correlation circle of the variables of the PLS components (Dim 1 and Dim2) is presented. Aroma, sweetness, and TSS displayed the highest correlations with overall liking. The TPA parameters were almost orthogonal in the PLS correlation plot, indicating that most of the differences found between the observations for those parameters did not cause variations in the overall liking scores of the assessors.

On the other hand, Figure 7 also highlights how the other TPA and colorimetric parameters did not contribute much to the correlations among the predictor and the dependent variable blocks.

## 4 Conclusions

Despite the sensorial, textural, colorimetric, and physicochemical differences found within the ST_brand and especially the LT_brand, it is clear from the results of univariate and multivariate assessments that the genotypic differences present among strawberry varieties did not lead to consistent variation in the overall liking scores of the assessors in this study. This is because the parameters that mainly contributed to product variability had a low impact on overall liking, as shown by the MFA and PLS assessments. The new methodology provides important information for warehouses and fruit producing organizations concerning strawberry commercialization that can be used to develop novel strategies. The results of this study suggest that a long-term brand is a viable solution to extend the length of the strawberry commercialization period, even though the brand is more susceptible to heterogeneity among quality attributes. Considering the results obtained in other similar trials considering other crop assessments [3], it is also clear that the viability of mixing varieties within a single brand is a crop-dependent issue. Therefore, each fruit species shall be submitted to this type of study in order to check the marketing strategy is viable. This study also highlights how many critical aspects of brand assessments and longitudinal studies can be handled by the use of appropriate models, such as mixed models, that can account for most of the information present in the data, as highlighted by the introduction of random terms in the univariate case and the use of a constrained model in the multivariate case. We suggest that the use of different covariance structures may be functional to improve the fit of ANOVA mixed models in sensory longitudinal studies, as described by this analysis, however, this type of model need to be available also in more user-friendly statistical software in order to become widespread at a research and industrial level. Our results also confirmed that differences among replicates within varieties should also be taken into account. However, our study has some limitations, such as the amount of data used, which should be expanded with samples from different harvesting years. We also suggest that our methodology should be applied at the consumer level to extend these findings to the population.

## Declarations

### Author contribution statement

Thais Mendes da Silva: Analyzed and interpreted the data; Wrote the paper.

Nicole Roberta Giuggioli: Performed the experiments; Contributed reagents, materials, analysis tools or data; Wrote the paper.

Cristiana Peano: Conceived and designed the experiments; Contributed reagents, materials, analysis tools or data.

### Funding statement

This research did not receive any specific grant from funding agencies in the public, commercial, or not-for-profit sectors.

### Data availability statement

Data will be made available on request.

### Declaration of interests statement

The authors declare no conflict of interest.

### Additional information

No additional information is available for this paper.
